# Small cardiac vein draining into the inferior vena cava

**DOI:** 10.1093/icvts/ivae162

**Published:** 2024-09-26

**Authors:** Filippos-Paschalis Rorris, Spyridoula Katsilouli, Dimitrios Bobos, Meletios Kanakis

**Affiliations:** Department of Pediatric and Adult Congenital Heart Surgery, Onassis Cardiac Surgery Center, Athens, Greece; Department of Computed Tomography, Onassis Cardiac Surgery Center, Athens, Greece; Department of Pediatric and Adult Congenital Heart Surgery, Onassis Cardiac Surgery Center, Athens, Greece; Department of Pediatric and Adult Congenital Heart Surgery, Onassis Cardiac Surgery Center, Athens, Greece

**Keywords:** Congenital heart disease, Anomalous cardiac vein, Inferior vena cava

## Abstract

Cardiac venous anomalies are rare congenital anatomical anomalies, which are most commonly found on computed tomography scans as an accidental finding. We report a case of a 14-year-old child who was operated for an atrial septal defect, and during the operation, we came across an anomalous drainage of the small cardiac vein into the inferior vena cava. The child’s postoperative course was uneventful.

## INTRODUCTION

The small cardiac vein is part of the greater cardiac venous system. It drains the inferior wall of the right ventricle, runs along with the right coronary artery in the right atrioventricular groove and ultimately empties into the coronary sinus [1]. It less often may drain directly into the right atrium or right ventricle [1]. Anomalous venous drainage of the cardiac venous system may have clinical implications in diagnostic and interventional procedures such as pacemaker lead implantation [1]. Anatomic anomalies of the venous system are most commonly discovered randomly [2]. Anomalous drainage of the small cardiac vein into the superior vena cava has been reported in the literature at least twice [3, 4]. However, to the best of our knowledge, this is the 1st reported case of the small cardiac vein draining into the inferior vena cava.

## CASE PRESENTATION

**Figure 1:**
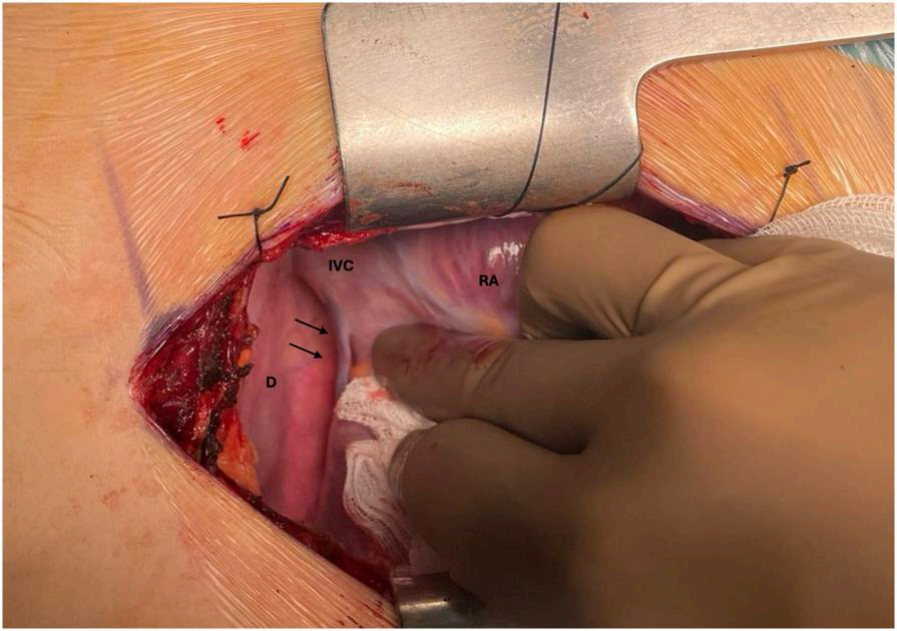
Intraoperative image demonstrating the anomalous small cardiac vein (arrows). D: diaphragm; IVC: inferior vena cava; RA: right atrium.

**Figure 2:**
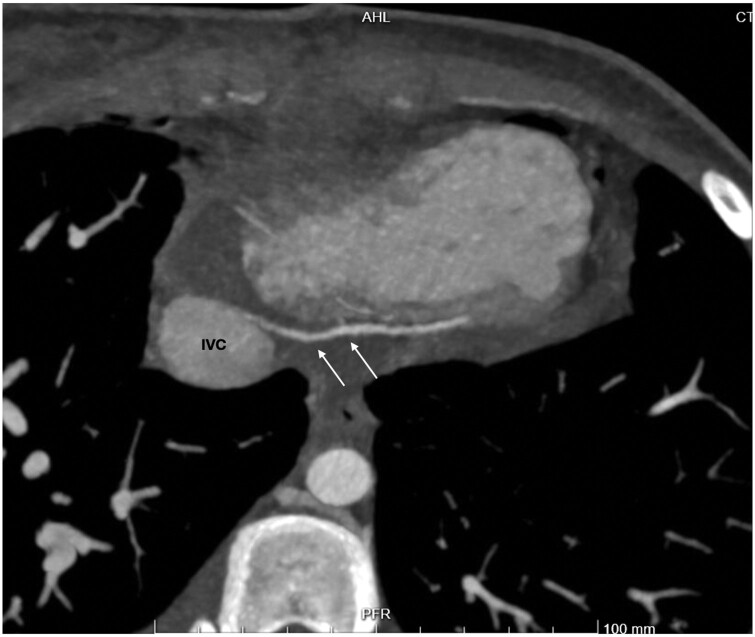
Computed tomography image demonstrating the anomalous small cardiac vein (arrows). IVC: inferior vena cava.

A 14-year-old child was referred to our Paediatric Heart Surgery centre for surgical management of a secundum atrial septal defect. Intraoperatively, during routine heart manipulation for bicaval cannulation, we noticed an unusual vessel running from the inferior ventricular wall of the right ventricle and draining into the inferior vena cava (Fig. 1). The child underwent the planned repair for the atrial septal defect. In order to clarify this rare finding, computed tomography angiography scan with targeted coronary vessel sequence was performed postoperatively (Fig. 2) after discussion and shared decision-making with the parents. The child’s postoperative course was unremarkable.

## DISCUSSION

We report an unusual finding of an anomalous small cardiac vein draining into the inferior vena cava, which was discovered during operation for an atrial septal defect in a child. The anomalous cardiac vein did not alter the procedure’s plan, but care had to be taken during inferior vena cava manipulation and purse string suturing of the inferior vena cava for bicaval cannulation. Small cardiac vein anomalous drainage has been reported in the literature but not in the inferior vena cava as of yet. Of note, the child had also right coronary artery high take-off from the aorta.

**Conflict of interest:** none declared.

## Data Availability

All data underlying this article are available in the article and there are no supplementary materials for this article.
